# Familial Exudative Retinopathy: A Case and Family Analysis

**DOI:** 10.4274/tjo.03185

**Published:** 2018-09-04

**Authors:** Hazan Gül Kahraman, Feray Koç, Nazife Sefi Yurdakul

**Affiliations:** 1 İzmir Atatürk Training and Research Hospital, Ophthalmology Clinic, İzmir, Turkey

**Keywords:** Retina, familial exudative vitreoretinopathy, genetics

## Abstract

Familial exudative vitreoretinopathy (FEVR) is a rare inherited disorder of retinal angiogenesis. A 49-year-old male patient was referred to our clinic for retinal vascular occlusion. His history, clinical findings, and fundus fluorescein angiography findings were evaluated. Family members were called and eye examinations were performed. Our patient was not born preterm and he reported decreased visual acuity after a traffic accident during childhood. He had laser treatment when he was 12 years old and again 1 month before our examination. He also had laser-assisted in situ keratomileusis surgery for both eyes in 2002. On examination, his visual acuity was 0.4 in the right eye and 0.3 in the left eye. He had cortical cataract in both eyes. Macula OCT revealed macular contour irregularity due to epiretinal membrane in his right eye and minimal perifoveal thinning in his left eye. On fundus photography, straightening of the retinal vessels, macular dragging, retinal folds on temporal retina, preretinal fibrosis, and laser spots were seen. FFA revealed avascular retinal areas with incomplete laser spots in the temporal, inferior, and superior parts of retina. He also had neovascularization with leakage in the temporal retina of his right eye. The patient’s brother, who was also born at full term, also had excessive branching of the vascular structures in the temporal peripheral retina, non-perfused cord vessels and avascular areas. In light of all these findings, we diagnosed our patient with Stage 2A FEVR and his brother with Stage 1 FEVR. In summary, FEVR is a clinically diagnosed disease. Because FEVR is inherited and potentially sight-threatening, family examination is helpful and important so that affected family members can be diagnosed and followed up.

## Introduction

Familial exudative vitreoretinopathy (FEVR) is a rare hereditary disease affecting retinal vascular development, and was first described in 1969 by Criswick and Schepens.^1^ The vascular pathologies were characterized by Canny and Oliver^2^ in 1976 using fundus fluorescein angiography (FFA). The primary clinical manifestation of the disease is an avascular peripheral retina. Subsequent ischemia may lead to other findings of FEVR, including serious problems such as neovascularization (NVE), fibrosis, posterior pole traction, retinal folds, and even retinal detachment.^3^ Diagnosis can be made based on the presence of these findings on fundus examination, but evaluation of family members can also support the diagnosis in advanced cases with complicated findings. Ranchod et al.^4^ and Pendergast and Trese.^5^ reported positive family history in 37% and 57.7% of patients with FEVR, respectively. In a study conducted in Turkey, 7 out of 10 patients were found to have a positive family history.^6^ In this report, we present the findings of a patient diagnosed with FEVR and another affected member of his family.

## Case Report

**Figure 1:**
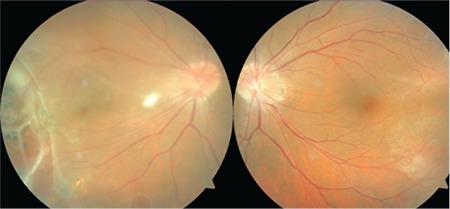
Fundus photographs of a patient with familial exudative vitreoretinopathy showing straightening of the temporal retinal vascular arcades, temporal dragging of the macula that was more prominent in the right eye, and laser spots with preretinal fibrosis in the right fundus temporal to the macula

A 49-year-old male patient was referred to our clinic with a prediagnosis of retinal vascular occlusion. In his medical history, he reported developing low vision after a traffic accident in childhood, having laser treatment in both eyes at 12 years of age and again 1 month earlier, and undergoing bilateral laser-assisted in situ keratomileusis in 2002. His medical and family histories were otherwise unremarkable. Following fundus examination and FFA, he was questioned again about his birth and he stated that he had been born full term by normal delivery. On ophthalmologic examination, his visual acuity was 0.4 in the right eye and 0.3 in the right eye, with mild cortical lens opacities. Fundus photography showed straightening of the temporal retinal vascular arcades and temporal dragging of the macula in both eyes (Figure 1). 

**Figure 2:**
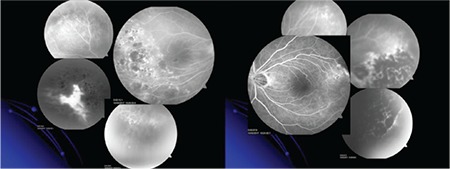
Fundus fluorescein angiography showing partially laser-treated avascular areas in the temporal retina of both eyes and leakage due to persistent retinal neovascularization in the right eye

Previously applied laser spots with corresponding preretinal fibrosis were observed in the temporal periphery. Although the nonperfused areas in the temporal retina had been partially laser treated, FFA revealed leakage due to persistent retinal NVE in the right eye (Figure 2).

**Figure 3:**
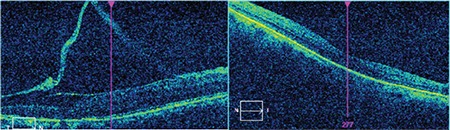
Macular optical coherence tomography showing disrupted macular contour associated with epiretinal membrane in the right macula and minimal perifoveal thinning in the left macula

Optical coherence tomography revealed disrupted macular contour associated with epiretinal membrane in the right macula and minimal perifoveal thinning in the left macula (Figure 3). 

**Figure 4:**
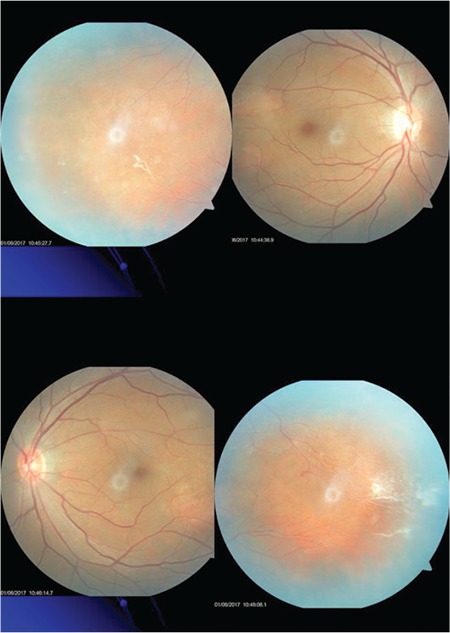
Color fundus photograph showing avascular areas, excessive vascular branching, and nonperfusing cord vessels in the temporal retinas of both eyes

Suspecting FEVR, the patient’s family members were invited for ophthalmologic examination. The patient’s father had normal ocular findings, while his brother showed straightening of the temporal vascular arcades in both eyes and excessive vascular branching and nonperfusing cord vessels in the peripheral vasculature, as well as temporal avascular areas (Figure 4).

His brother was also not born prematurely. FFA was not performed for his brother because he did not return for the procedure. FFA in the patient’s father was within normal limits. Based on the brother’s findings and the revised Pendergast and Trese^7^ classification, the patient was diagnosed with stage 2A FEVR and his brother was diagnosed with stage 1A FEVR (Table 1). The patient’s other family members did not appear for examination. Additional laser therapy to the nonperfused areas was recommended due to persistent NVE. When obtaining his family history, it was learned that his parents were second-degree cousins.

## Discussion

FEVR is diagnosed clinically. In the early stages, it is important to differentiate FEVR from retinopathy of prematurity. However, in older patients presenting with findings such as proliferative vitreoretinopathy (PVR), retinal detachment, and vitreous hemorrhage, especially those with asymmetric involvement, pathologies such as Coats disease, Eales disease, retinosis, sickle cell anemia, and toxocariasis must be excluded. Patients may also present with various combinations of macular dragging, radial retinal folds, NVE, preretinal vitreous organization, vitreoretinal proliferation, subretinal exudation, and retinal detachment. PVR is more common in advanced disease, and requires multiple surgical interventions.^8^

Milder forms of FEVR may manifest with peripheral avascularity accompanied by vitreoretinal adhesions, veno-venous anastomoses, increased vascular branching, and V-shaped retinochoroidal degeneration.^9^ Some FEVR patients who are asymptomatic with approximately normal fundus examination are reported to have smaller optical disc diameter, greater optic disc-macular distance/optic disc diameter ratio, and higher vessel density from the optic disc compared to a control group.^10^ We also observed these findings in the patient’s brother. These findings may support a diagnosis of FEVR in suspected cases.

To date, mutations in 5 genes have been associated with the AEVR phenotype (*NDP, FZD4, LRP5, TSPAN12, ZNF408*).^11,12^ Autosomal recessive, autosomal dominant, and X-related inheritance patterns have been reported. Therefore, while patients may have a positive family history, a negative family history may not be significant because family members can exhibit different clinical severity. One of the main challenges to diagnosis is that the patient’s parents are often healthy. The examination of other family members, especially siblings, is important to prevent further suffering associated with the disease.^13^ Because FEVR carries the risk of blindness, screening family members not only facilitates diagnosis, but is also key for follow-up and future genetic counseling for these patients. In addition, evaluating family members can expand our knowledge of the clinical course of the various phenotypes of this rare condition. Our patient had undergone laser therapy at another center and was undiagnosed when he was referred to our clinic. His family members were evaluated for this purpose for the first time and FEVR was detected in his brother. Because we could not perform genetic analysis in this case, the affected gene and inheritance pattern could not be determined, but the patient’s family was made aware of the possible complications and genetic transmission of this disease.

FEVR can present in older patients. Family screening assists diagnosis and also enables affected individuals to be identified before becoming symptomatic. These individuals can be informed about and followed for the possible complications of the disease. Furthermore, inheritance pattern may be determined based on the affected family members and genetic counseling can be provided for future generations.

## Figures and Tables

**Table 1:**
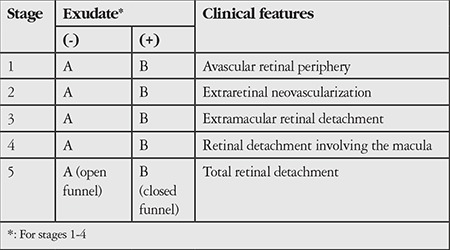
Updated clinical classification of familial exudative vitreoretinopathy
